# Sodium Intake and Its Relation to Coronary Calcium Score in Relatively Young Korean Adults

**DOI:** 10.5334/gh.1528

**Published:** 2026-02-16

**Authors:** Sung Keun Park, Yeongu Chung, Chang-Mo Oh, Ju Young Jung

**Affiliations:** 1 Center for Cohort Studies, Total Healthcare Center, Kangbuk Samsung Hospital, Sungkyunkwan University School of Medicine, Seoul, Republic of Korea; 2 Department of Neurosurgery, Kangbuk Samsung Hospital, SungKyunkwan University School of Medicine, Seoul, Republic of Korea; 3 Department of Preventive Medicine, School of Medicine, Kyung Hee University, Seoul, Republic of Korea; 4 Total Healthcare Center, Kangbuk Samsung Hospital, Sungkyunkwan University School of Medicine, Seoul, Republic of Korea

**Keywords:** sodium intake, coronary artery calcium score, coronary artery disease

## Abstract

**Background:**

There have been inconsistent findings regarding the effects of high sodium intake on coronary artery disease (CAD). This study therefore aimed to investigate the relationship between sodium intake and the coronary artery calcium score (CACS), reflecting coronary atherosclerosis.

**Method:**

Study participants were 89,337 Koreans in whom CACS and dietary sodium intake were measured during a health check-up. They were grouped according to quartile levels of dietary sodium intake. Multivariable logistic regression analysis was performed to evaluate the association between sodium intake quartiles and CACS > 0 (adjusted odds ratio [95% confidence interval]). Among participants with CACS > 0, linear regression analysis was conducted to assess whether a dose–response relationship exists between sodium intake and the severity of coronary artery calcification.

**Results:**

In all of the participants, an increase in sodium intake was modestly associated with CACS > 0 (first quartile: reference, second quartile: 1.06 [1.00–1.14], third quartile: 1.09 [1.01–1.16], and fourth quartile: 1.11 [1.03–1.20]). This association was observed only in men (first quartile: reference, second quartile: 1.07 [1.00–1.14], third quartile: 1.08 [1.01–1.16], and fourth quartile: 1.10 [1.02–1.19]). However, women did not show a significant association. Linear regression analysis also failed to show a significant association between quartile levels of sodium intake and CACS.

**Conclusion:**

There was a positive association between dietary sodium intake and a CACS > 0 in men. Our results suggest one possible mechanism linking high sodium intake to CAD.

## Introduction

Sodium (Na) is an essential nutrient that plays a critical role in maintaining physiological homeostasis. It regulates fluid balance, acid–base equilibrium, and osmotic pressure and is essential for nerve impulse transmission and muscle contraction (1,2). However, there has been growing concern regarding the adverse effects of excessive sodium intake on cardiovascular health. Experimental and epidemiological evidence suggests that high sodium intake contributes to fluid retention, sympathetic nervous system activation, endothelial dysfunction, and arterial stiffness, which collectively promote the development of hypertension (3). In line with these mechanisms, higher sodium intake has been associated with an increased risk of cardiovascular disease (CVD) (4,5).

Coronary artery disease (CAD) is a life-threatening CVD. Statistics indicated that there were 315 million prevalent cases of CAD globally in 2022 (6). Although high sodium intake is widely believed to have a detrimental effect on CAD, evidence directly linking sodium intake to CAD is inconsistent (7,8,9,10,11,12). Moreover, relatively few studies have evaluated the relationship between sodium intake and subclinical coronary atherosclerosis.

The coronary artery calcium score (CACS), assessed by computed tomography, quantifies calcified plaque burden in the coronary arteries and is a well-established surrogate marker of coronary atherosclerosis (13). CACS has been shown to improve risk stratification for CAD and to predict future cardiovascular events in asymptomatic populations (14,15,16). Compared with clinical endpoints like CAD, CACS allows for the assessment of subclinical coronary atherosclerosis before the onset of overt disease, providing a useful intermediate marker for investigating potential associations with modifiable risk factors such as dietary sodium intake.

In the present study, we investigated the association between levels of dietary sodium intake and CACS > 0. In addition, among individuals with CACS > 0, the linear relationship between sodium intake and CACS was analyzed to identify whether a dose–response relationship exists between dietary sodium intake and the severity of coronary artery calcification.

## Methods

### Study participants and exclusion criteria

**Figure 1:**
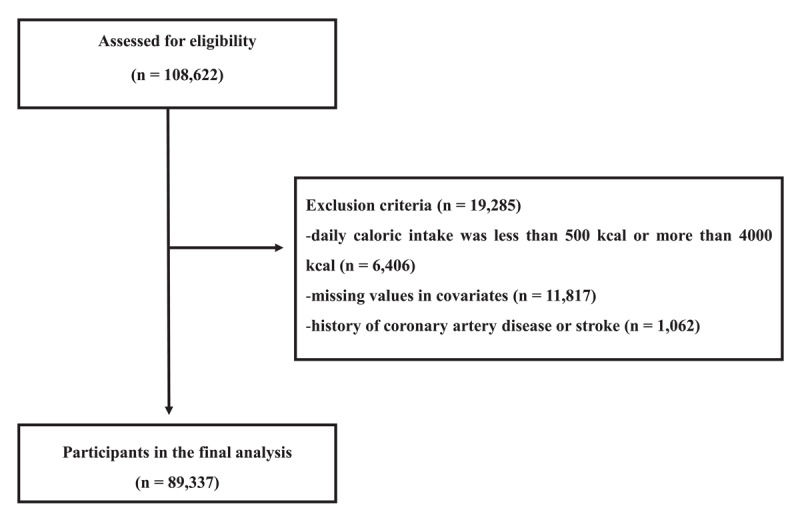
Flow chart of enrolled study participants.

We used the data collected from the Kangbuk Samsung Health Study (KSHS). The KSHS is an ongoing large-scale cohort study conducted at Kangbuk Samsung Hospital, Korea. Study participants were employees and their families who had regular health check-ups. Among the KSHS cohort participants, this study initially included 108,622 who responded to the Food Frequency Questionnaire (FFQ) and selected multi-detector computed tomography (MDCT) as a medical check-up option between March 2011 and December 2019. In the case of participants who had undergone several health check-ups, the first visit was included. We excluded 19,285 participants with inappropriate calorie intake, missing values in covariates, and a history of stroke or CAD from the final analysis (Figure 1). The final number of participants included in the analysis was therefore 89,337.

### Clinical, health-related behavior, and MDCT data assessment

All participants responded to questionnaires about health-related behaviors (drinking, smoking, and physical activity), socioeconomic factors (income, marriage, and education), medical history, and medication use. Blood samples were collected after more than 12 hours of fasting and were drawn from an antecubital vein. The collected blood was processed in an automated laboratory to obtain biochemical data such as fasting blood sugar, HbA1c, and low-density lipoprotein (LDL)-cholesterol.

An MDCT scan was performed by a LightSpeed VCT XTe-64 slice MDCT scanner (GE Healthcare, Tokyo, Japan). The inter-observer reliability and intra-observer reliability for the CACS were both excellent (intraclass correlation coefficient = 0.99). Details of CACS and other data collection methods have been described in a previous study (17,18).

### Dietary data assessment

We assessed the dietary sodium intake of KSHS participants using the FFQ that was developed for the Korean genome epidemiologic study. The Korean genome epidemiologic study is a cohort study led by a Korean government agency that collected health-related data from the general Korean population (19). Dietary items for the Korean version of the FFQ were obtained from the Korea Health and Nutrition Examination Survey (20,21). A detailed description of the FFQ (20) and its validation in the Korean population have been described in a previous study (21). A previous study from our group confirmed that sodium intake was assessed by this FFQ, and the calculation method was positively correlated with the salting habit score (22). Total energy and nutrient intake were calculated by the CAN-Pro 3.0 software developed by the Korean Nutrition Society (23).

### Statistical analysis method

All participants and the sex subgroup were divided into quartile groups according to their daily sodium intake. The main variables in this quartile group were expressed as means ± standard deviation (SD) for continuous variables and as proportions (%) for categorical variables. Main clinical, demographic, and biochemical dietary parameters between the four groups were compared using ANOVA (continuous variables) or the chi-square test (categorical variables). Given the differences in the prevalence of coronary artery calcification between men and women, we also performed sex subgroup analyses.

CACS was categorized as 0 and >0. Previous meta-analyses have demonstrated that CACS > 0 is associated with a significantly increased risk of major adverse cardiovascular and cerebrovascular events as well as all-cause mortality compared with CACS = 0 (13). Given the highly right-skewed distribution of CACS and the substantial proportion of zero values, CAC presence was analyzed as a binary outcome. The logistic regression analysis was used to investigate the association between dietary sodium intake and CACS > 0 to identify whether increased sodium intake was associated with the presence of coronary artery calcification. In the logistic regression analysis, an unadjusted model and three adjusted models were used to obtain odds ratios (ORs) and 95% confidence intervals (95% CIs) in each study group (adjusted OR [95% CI]). Covariates were selected based on known associations with both dietary pattern (e.g., age, sex, education, diabetes mellitus (DM), alcohol intake, body mass index (BMI)) and coronary artery calcification (e.g., age, sex, BMI, smoking, systolic blood pressure (BP), anti-hypertensive medication, DM, physical activity, LDL-cholesterol, lipid-lowering medication), to minimize residual confounding. Among the three adjusted models, Model 1 included total calorie intake, and Model 2 included total calorie intake, demographic factors (sex, age), health-related behaviors (alcohol intake, smoking, physical activity), and socioeconomic factors (high education). Model 3 was adjusted for all possible variables, including underlying diseases and medication history, which may affect coronary artery calcification (Model 3: total calorie intake, age, sex, alcohol intake, smoking, education, physical activity, systolic BP, anti-hypertensive medication, DM, BMI, LDL-cholesterol, lipid-lowering medication). The fully adjusted model (Model 3) included all covariates and served as the primary reference in the present study. Trend analysis was performed based on the median sodium intake of each sodium intake quartile. To verify multicollinearity between variables, we analyzed the variance inflation factor (VIF), and it was confirmed that there were no variables with a VIF greater than 10. Model discrimination was assessed using C-statistics.

In addition, among participants with CACS > 0, linear regression analyses were conducted to examine the dose–response relationship between dietary sodium intake and the severity of coronary artery calcification. Non-standardized correlation coefficients (β) and P-values of sodium intake (mg/day) were obtained in the unadjusted and three adjusted models. All statistical analyses were performed using R 4.5.0 (R Foundation for Statistical Computing, Vienna, Austria), and a two-sided P-value of <0.05 (two-sided) was considered statistically significant in all analyses.

## Result

Table 1 presents the clinical characteristics of the study participants in relation to quartile levels of dietary sodium intake. The study participants featured a preponderance of men (n = 69,086, 77.3%) and a relatively young age. Sodium intake increased with the proportion of men, alcohol use, smoking, BP, LDL-cholesterol, physical activity, and total calorie intake. The prevalence of hypertension and DM also increased with the sodium intake quartile. While there was no significant difference in CACS among quartile groups, the proportion of CACS > 0 increased with quartile levels of sodium intake. The clinical characteristics of the men and women are presented in Supplementary Tables 1 and 2, respectively.

**Table 1:** Baseline clinical characteristics of study participants according to dietary sodium intake.


CHARACTERISTICS	QUARTILE 1	QUARTILE 2	QUARTILE 3	QUARTILE 4	P VALUE

number	22,335	22,335	22,334	22,333	

Men (n, [%])	16,271 (72.8%)	16,918 (75.7%)	17,718 (79.3%)	18,179 (81.4%)	<0.001

Age (year)	40.5 ± 8.2	40.2 ± 7.9	40.3 ± 8.0	40.7 ± 8.0	<0.001

BMI (kg/m^2^)	24.1 ± 3.3	24.3 ± 3.3	24.6 ± 3.3	24.9 ± 3.4	<0.001

LDL-cholesterol (mg/dl)	128.7 ± 32.6	129.2 ± 32.4	129.5 ± 32.4	129.4 ± 32.3	0.043

Average alcohol use (g/day)	12.4 ± 18.5	14.4 ± 20.3	16.3 ± 22.5	19.7 ± 27.5	<0.001

Current smoker (%)	17.3%	20.6%	23.4%	26.9%	<0.001

High physical activity (%)	15.1%	16.2%	17.2%	19.1%	<0.001

High education (%)	69.8%	71.5%	72.3%	70.1%	<0.001

Hypertension (%)	14.5%	14.7%	16.2%	17.0%	<0.001

Systolic blood pressure (mmHg)	111.7 ± 12.4	112.2 ± 12.4	112.8 ± 12.3	113.6 ± 12.4	<0.001

Diastolic blood pressure (mmHg)	72.3 ± 9.8	72.5 ± 9.8	72.9 ± 9.7	73.5 ± 9.8	<0.001

Anti-hypertensive medication (%)	6.3%	6.3%	7.2%	7.4%	<0.001

DM (%)	4.9%	5.3%	5.4%	6.0%	<0.001

Total calorie intake (kcal/day)	1074.6 ± 355.3	1336.5 ± 407.5	1581.6 ± 470.0	1955.8 ± 631.4	<0.001

Dietary sodium intake (mg/day)	640.8 ± 227.1	1214.7 ± 152.8	1844.2 ± 224.3	3271.8 ± 974.4	<0.001

Lipid lowering medication (%)	3.8%	4.2%	4.2%	4.5%	0.006

CAC score	8.9 ± 74.7	9.2 ± 75.6	8.9 ± 65.7	9.4 ± 67.2	0.888

CAC score >0 (n, [%])	2,539 (11.4%)	2,630 (11.8%)	2,760 (12.4%)	2,955 (13.2%)	<0.001


Continuous variables are expressed as mean (±SD), and categorical variables are expressed as number (percentage (%)). BMI: body mass index, DM: diabetes mellitus, LDL: low-density lipoprotein, CAC: Coronary Calcium Score.

In Table 2, unadjusted and multivariable-adjusted logistic regression analysis indicated that increased sodium intake was associated with a slight increase in the likelihood of having CACS > 0 across all quartile groups (Model 3: OR [95% CI] for the highest vs. lowest quartile: 1.11 [1.03–1.20], P for trend = 0.010). This association was significant only in men (OR [95% CI]: 1.10 [1.02–1.19], P for trend = 0.034), while no significant association was observed in women (OR [95% CI]: 1.11 [0.88–1.39], P for trend = 0.284). The full details of the regression estimates are provided in Table 2.

**Table 2:** Odds Ratio (OR) and 95% confidence intervals (CI) for CAC score >0 according to the quartile groups of dietary sodium intake.


	QUARTILE 1	QUARTILE 2	QUARTILE 3	QUARTILE 4	P FOR TREND

**All participants (n)**	22,335	22,335	22,334	22,333	

Unadjusted OR	1.00 (Reference)	1.04 (0.98–1.10)	1.10 (1.04–1.16)	1.19 (1.12–1.26)	<0.001

Model 1	1.00 (Reference)	1.08 (1.02–1.14)	1.18 (1.11–1.25)	1.34 (1.25–1.43)	<0.001

Model 2	1.00 (Reference)	1.09 (1.02–1.16)	1.11 (1.04–1.19)	1.14 (1.06–1.23)	0.002

Model 3	1.00 (Reference)	1.06 (1.00–1.14)	1.09 (1.01–1.16)	1.11 (1.03–1.20)	0.010

Case [n, (%)]	2,539 (11.4%)	2,630 (11.8%)	2,760 (12.4%)	2,955 (13.2%)	

Sodium intake (median) (mg/day)	≤ 956 (681)	956–1488 (1211)	1488–2275 (1828)	≥ 2275 (2989)	

**Men (n)**	17,277	17,269	17,269	17,271	

Unadjusted OR	1.00 (Reference)	1.01 (0.95–1.07)	1.03 (0.97–1.09)	1.07 (1.00–1.13)	0.028

Model 1	1.00 (Reference)	1.09 (1.02–1.16)	1.18 (1.11–1.26)	1.35 (1.26–1.45)	<0.001

Model 2	1.00 (Reference)	1.08 (1.01–1.15)	1.10 (1.03–1.18)	1.13 (1.04–1.22)	0.005

Model 3	1.00 (Reference)	1.07 (1.00–1.14)	1.08 (1.01–1.16)	1.10 (1.02–1.19)	0.034

Case [n, (%)]	2,409 (13.9%)	2,431 (14.1%)	2,463 (14.3%)	2,542 (14.7%)	

Range of intake (median) (mg/day)	≤ 988 (703)	988–1534 (1251)	1534–2327 (1879)	≥ 2327 (3048)	

**Women (n)**	8,019	7,987	8,007	7,994	

Unadjusted OR	1.00 (Reference)	0.99 (0.82–1.20)	1.19 (0.99–1.43)	1.41 (1.19–1.68)	<0.001

Model 1	1.00 (Reference)	1.02 (0.84–1.23)	1.25 (1.03–1.51)	1.55 (1.26–1.90)	<0.001

Model 2	1.00 (Reference)	1.05 (0.85–1.30)	1.12 (0.91–1.38)	1.13 (0.90–1.41)	0.313

Model 3	1.00 (Reference)	0.98 (0.78–1.21)	1.08 (0.87–1.34)	1.11 (0.88–1.39)	0.284

Case [n, (%)]	228 (4.5%)	226 (4.5%)	269 (5.3%)	316 (6.2%)	

Range of intake (median) (mg/day)	≤ 867 (617)	867–1334 (1090)	1334–2064 (1637)	≥ 2064 (2779)	


Model 1: total calorie intake. Model 2: total calorie intake, age, sex, alcohol intake, smoking, education, physical activity. Model 3: total calorie intake, age, sex, alcohol intake, smoking, education, physical activity, systolic blood pressure, anti-hypertensive medication, DM, BMI, LDL cholesterol, lipid lowering medication. (sex excluded in gender subgroup analysis).

Linear regression analysis was conducted to estimate the linear relationship between sodium intake and CACS in participants with a CACS > 0 (Table 3). Unlike logistic regression analysis, which showed a positive association between sodium intake and CACS > 0 in all participants, linear regression analysis failed to show a significant linear relationship between sodium intake and CACS in all study participants. Sex subgroup analysis also did not show a significant relationship between sodium intake and CACS in both men and women.

**Table 3:** The relationships between sodium intake and CAC score.


	UNADJUSTED		MODEL 1		MODEL 2		MODEL 3	
			
	β(SE)	P VALUE	β(SE)	P VALUE	β(SE)	P VALUE	β(SE)	P VALUE

**All participants**	–0.002 (0.002),	0.193	0.001 (0.002),	0.738	–0.002 (0.002),	0.319	–0.002 (0.002),	0.177

**Men**	–0.002 (0.002),	0.334	0.001 (0.002),	0.470	–0.001 (0.002),	0.539	–0.002 (0.002),	0.334

**Women**	–0.006 (0.004),	0.198	–0.007 (0.005),	0.214	–0.008 (0.005),	0.132	–0.008 (0.005),	0.107


Values in table are non-standardized correlation coefficients (β) and p values of sodium intake (mg/day). SE: Standard error. Model 1: total calorie intake. Model 2: total calorie intake, age, sex, alcohol intake, smoking, education, physical activity. Model 3: total calorie intake, age, sex, alcohol intake, smoking, education, physical activity, systolic blood pressure, anti-hypertensive medication, DM, BMI, LDL cholesterol, lipid lowering medication. (sex excluded in gender subgroup analysis).

## Discussion

In a cohort of 89,337 Korean adults, sodium intake ≥ quartile 2 was more significantly associated with CACS > 0 compared with quartile 1, even after adjusting for conventional risk factors for CAD, including BP, hypertension, DM, and LDL-cholesterol. This finding indicates that high sodium intake is independently associated with an increase in CACS. Considering the potential link between CACS, coronary atherosclerosis, and CAD, this result supports those of previous studies that have shown a detrimental effect of high sodium intake on CAD. Observational studies have demonstrated that an elevation in baseline urinary sodium excretion was associated with the increased risk of CAD (7,8). In overweight individuals, 100 mmol higher sodium intake per day was associated with a 44% increase in coronary heart disease mortality (9). However, inconsistent results have questioned the positive association between sodium intake and CAD. Cohort studies in Europeans showed that increased urinary sodium excretion did not predict coronary heart disease or related mortality (10,11). A former prospective study showed that an increase in usual sodium intake was not associated with an increased risk for mortality of ischemic heart disease (12). Moreover, several studies have demonstrated that low sodium intake was associated with an increased risk of CAD and its mortality (24,25,26).

To better understand the effect of sodium intake on CAD, it is useful to investigate the relationship between sodium intake and surrogates of coronary atherosclerosis before the onset of overt CAD. CACS quantifies the extent of calcified atherosclerotic plaque in the coronary arteries and serves as a surrogate marker of overall coronary atherosclerotic burden. However, published data on the relationship between sodium intake and CACS are limited. Only one recent study showed that an increase in 24-hour urinary sodium excretion was associated with a 16% increase in higher CACS with minimal adjustment (27). Therefore, by using CACS as the dependent variable, our study will provide novel insight into the association between dietary sodium intake and subclinical coronary atherosclerosis and may offer mechanistic explanations for the effect of high sodium intake on traditional clinical endpoints of CAD. The mechanism linking sodium intake to coronary artery calcification may involve elevation of BP (28,29,30,31), arterial stiffness, and endothelial dysfunction driven by high sodium intake (32,33,34). However, evidence is still insufficient to explain the mechanism underlying the association between sodium intake and CACS. Further studies should be conducted to elucidate the underlying mechanism.

In our analysis, despite the positive association between sodium intake and CACS > 0, the magnitude of association was modest, with a 6–10% increased OR across quartile groups. Therefore, it is less likely that the effect of sodium on CAD surpasses that of classic risk factors, including BP, dyslipidemia, obesity, and age. A recent study also showed that adjustment for BP negates the significant association between sodium excretion and higher CACS and coronary artery stenosis observed with minimal adjustment (27). In addition, it is presumed that there are individuals more susceptible to the effects of sodium on CAD. Joosten et al. demonstrated that an increase in sodium excretion was associated with the risk of CAD only in subjects with hypertension and elevated plasma N-terminal pro-B-type natriuretic peptide (35). Nonetheless, it should be careful not to overlook the association between sodium intake and CACS, even if it is weak in our analysis. This modest degree of association does not necessarily suggest that high sodium intake has little effect on the risk of CAD. In particular, it is considered that most of our study participants are relatively young and apparently healthy. It takes time for atherosclerosis of the coronary artery to generate a CACS of >0. Thus, it is probable that CACS > 0 is yet to manifest in young adults with high sodium intake, despite atherosclerotic changes in the coronary artery. Moreover, considerable time is required for atherosclerosis as reflected by CACS to progress into overt CAD. In young adults with existing CACS > 0, the adverse effects of high sodium intake on the coronary artery may accumulate and, in turn, put them at an increased risk of CAD. There is a need for clinical interest and continuing studies on the harmful effects of high sodium intake on the coronary arteries.

In our study, multivariate regression analysis demonstrated a positive association between dietary sodium intake and CACS > 0, whereas linear regression analysis failed to reveal a significant association between sodium intake and CACS among individuals with CACS > 0. The characteristics of our study participants may be one explanation for this discrepancy. Our study participants were composed of relatively young and apparently healthy individuals; thus, even among those with CACS > 0, cases of severe coronary calcification with high CACS were likely to be rare. Indeed, the mean CACS ranged from 8.9 to 9.4 across all quartile groups, indicating a generally low burden of coronary calcification in our study participants. Another possible explanation is that individuals with high CACS may have already been excluded at baseline due to the presence of established CAD or the occurrence of various adverse cardiovascular outcomes. Finally, the potential influence of residual confounding should be considered because our study could not account for all variables that affect the relationship between sodium intake and CACS.

It appears that there is a sex-specific difference in the association between sodium intake and CACS. In the present study, women failed to show any association between sodium intake and CACS as opposed to men. Plausible explanations for this finding may be better conditions in metabolic profiles and health behaviors in women. Our analysis showed that women had lower levels of LDL-cholesterol, BP, hypertension, DM, smoking, and alcohol intake than men. These variables act as cardiovascular risk factors that can generate synergy with high sodium intake during the progression of coronary atherosclerosis. Preferable metabolic and behavioral conditions in women may have a protective effect against coronary atherosclerosis. In addition, sex differences in cardiovascular risk should be considered. Premenopausal women are more immune to CVD than postmenopausal women and men. Estrogen plays a crucial role in the cardioprotective properties of premenopausal women. Estrogen mediates its cardioprotective actions by increasing angiogenesis and vasodilation and decreasing reactive oxygen species, oxidative stress, and fibrosis (36). Considering the mean age of our study participants, most of the female subjects were premenopausal. Therefore, it is presumed that favorable clinical conditions and hormonal features in women attenuate the influence of high sodium intake on CACS.

The present study has several limitations that deserve to be mentioned. First, the results were derived from a cross-sectional design and observational analysis, which cannot provide any information about causative relationships. Second, most study participants were relatively young and healthy adults. Thus, it is unlikely that our results can be generalized to the clinical population and the elderly. Third, it is difficult to consider all confounding factors involved in the association between sodium intake and CACS. We cautiously acknowledge the possibility that residual confounding may have influenced our results. Fourth, dietary sodium intake was assessed using a self-reported questionnaire, which is inherently subject to recall bias. This limitation may lead to measurement error in sodium intake.

In conclusion, the present study demonstrated that increased sodium intake was modestly associated with CACS > 0 among men. This finding may serve as evidence linking high sodium intake to an increased risk of CAD among men. However, it should be recognized that there was no linear relationship between sodium intake and CACS. Moreover, women did not show any significant associations. Further studies should be conducted to investigate the effect of sodium intake on CAD through longitudinal analysis with diverse populations and covariates.

## Data Accessibility Statement

The data that support the findings of this study are available from the Kangbuk Samsung Cohort Study, but restrictions apply to the availability of these data, which were used under license for the current study and so are not publicly available. Data are, however, available from the authors upon reasonable request and with permission of the Kangbuk Samsung Cohort Study.

## Additional File

The additional file for this article can be found as follows:

10.5334/gh.1528.s1 Supplementary File. Supplementary Tables 1 and 2.

## References

[1] Kotchen TA, Cowley AW Jr, Frohlich ED. Salt in health and disease—a delicate balance. N Engl J Med. 2013;368:1229–1237. DOI: 10.1056/NEJMra1212606 23534562

[2] Glynn IM. Sodium and potassium movements in human red cells. J Physiol. 1956;134:278–310. DOI: 10.1113/jphysiol.1956.sp005643 13398911 PMC1359203

[3] Grillo A, Salvi L, Coruzzi P, Salvi P, Parati G. Sodium intake and hypertension. Nutrients. 2019;11:1970. DOI: 10.3390/nu11091970 31438636 PMC6770596

[4] Wang YJ, Yeh TL, Shih MC, Tu YK, Chien KL. Dietary sodium intake and risk of cardiovascular disease: A systematic review and dose-response meta-analysis. Nutrients. 2020;12:2934. DOI: 10.3390/nu12102934 32992705 PMC7601012

[5] Ma Y, He FJ, Sun Q, Yuan C, Kieneker LM, Curhan GC, et al. 24-hour urinary sodium and potassium excretion and cardiovascular risk. N Engl J Med 2022;386:252–263. DOI: 10.1056/NEJMoa2109794 34767706 PMC9153854

[6] Stark B, Johnson C, Roth G. Global prevalence of coronary artery disease: an update from the global burden of disease study. JACC. 2024;83(13_Supplement):2320. DOI: 10.1016/S0735-1097(24)04310-9

[7] O’Donnell MJ, Yusuf S, Mente A, Gao P, Mann JF, Teo K, et al. Urinary sodium and potassium excretion and risk of cardiovascular events. JAMA. 2011;306:2229–2238. DOI: 10.1001/jama.2011.1729 22110105

[8] Tuomilehto J, Jousilahti P, Rastenyte D, Moltchanov V, Tanskanen A, Pietinen P, et al. Urinary sodium excretion and cardiovascular mortality in Finland: A prospective study. Lancet. 2001;357:848–851. DOI: 10.1016/S0140-6736(00)04199-4 11265954

[9] He J, Ogden LG, Vupputuri S, Bazzano LA, Loria C, Whelton PK. Dietary sodium intake and subsequent risk of cardiovascular disease in overweight adults. JAMA. 1999;282:2027–2034. DOI: 10.1001/jama.282.21.2027 10591385

[10] Tunstall-Pedoe H, Woodward M, Tavendale R, A’Brook R, McCluskey MK. Comparison of the prediction by 27 different factors of coronary heart disease and death in men and women of the Scottish Heart Health Study: Cohort study. BMJ. 1997;315:722–729. DOI: 10.1136/bmj.315.7110.722 9314758 PMC2127508

[11] Geleijnse JM, Witteman JC, Stijnen T, Kloos MW, Hofman A, Grobbee DE. Sodium and potassium intake and risk of cardiovascular events and all-cause mortality: The Rotterdam study. Eur J Epidemiol. 2007;22:763–770. DOI: 10.1007/s10654-007-9186-2 17902026 PMC2071962

[12] Yang Q, Liu T, Kuklina EV, Flanders WD, Hong Y, Gillespie C, et al. Sodium and potassium intake and mortality among US adults: Prospective data from the Third National Health and Nutrition Examination Survey. Arch Intern Med. 2011;171:1183–1191. DOI: 10.1001/archinternmed.2011.257 21747015

[13] Shreya D, Zamora DI, Patel GS, Grossmann I, Rodriguez K, Soni M, et al. Coronary artery calcium score – A reliable indicator of coronary artery disease? Cureus. 2021;13:e20149. DOI: 10.7759/cureus.20149 35003981 PMC8723785

[14] Khan SS, Post WS, Guo X, Tan J, Zhu F, Bos D, et al. Coronary artery calcium score and polygenic risk score for the prediction of coronary heart disease events. JAMA. 2023;329:1768–1777. DOI: 10.1001/jama.2023.7575 37219552 PMC10208141

[15] Greenland P, LaBree L, Azen SP, Doherty TM, Detrano RC. Coronary artery calcium score combined with Framingham score for risk prediction in asymptomatic individuals. JAMA. 2004;291:210–215. DOI: 10.1001/jama.291.2.210 14722147

[16] Raggi P, Callister TQ, Cooil B, He ZX, Lippolis NJ, Russo DJ, et al. Identification of patients at increased risk of first unheralded acute myocardial infarction by electron-beam computed tomography. Circulation. 2000;101:850–855. DOI: 10.1161/01.cir.101.8.850 10694523

[17] Park SK, Kang JG, Seok HS, Jung JY. Echocardiographic parameters of left ventricular structure and diastolic function and their relation to coronary artery calcification. Int J Cardiovasc Imaging. 2021;37:2861–2869. DOI: 10.1007/s10554-021-02256-6 33945053

[18] Jung JY, Oh CM, Kim E, Park SK. Dietary sodium intake and its relation to sleep duration, sleep quality and nocturnal urination in working-aged Korean adults. Nutr Bull. 2023;48(3):365–375. DOI: 10.1111/nbu.12629 37458133

[19] Kim Y, Han BG; KoGES Group. Cohort profile: The Korean genome and epidemiology study (KoGES) consortium. Int J Epidemiol. 2017;46:e20. DOI: 10.1093/ije/dyv316 27085081 PMC5837648

[20] Ahn Y, Lee JE, Paik HY, Lee HK, Jo I Kimm K. Development of a semi-quantitative food frequency questionnaire based on dietary data from the Korea National Health and Nutrition Examination Survey. Nutr Sci. 2003;6:173–184.

[21] Ahn Y, Kwon E, Shim JE, Park MK, Joo Y, Kimm K, et al. Validation and reproducibility of food frequency questionnaire for Korean genome epidemiologic study. Eur J Clin Nutr. 2007;61(12):1435–1441. DOI: 10.1038/sj.ejcn.1602657 17299477

[22] Choi Y, Lee JE, Chang Y, Kim MK, Sung E, Shin H, et al. Dietary sodium and potassium intake in relation to non-alcoholic fatty liver disease. Br J Nutr. 2016;116:1447–1456. DOI: 10.1017/S0007114516003391 27725000

[23] The Korean Nutrition Society. CAN-Pro 3.0 software; 2005.

[24] Alderman MH, Madhavan S, Cohen H, Sealey JE, Laragh JH. Low urinary sodium is associated with greater risk of myocardial infarction among treated hypertensive men. Hypertension. 1995;25:1144–1152. DOI: 10.1161/01.hyp.25.6.1144 7768554

[25] Cohen HW, Hailpern SM, Fang J, Alderman MH. Sodium intake and mortality in the NHANES II follow-up study. Am J Med. 2006;119:275.e7–14. DOI: 10.1016/j.amjmed.2005.10.042 16490476

[26] Stolarz-Skrzypek K, Kuznetsova T, Thijs L, Tikhonoff V, Seidlerová J, Richart T, et al. Fatal and nonfatal outcomes, incidence of hypertension, and blood pressure changes in relation to urinary sodium excretion. JAMA. 2011;305:1777–1785. DOI: 10.1001/jama.2011.574 21540421

[27] Wuopio J, Ling YT, Orho-Melander M, Engström G, Ärnlöv J. The association between sodium intake and coronary and carotid atherosclerosis in the general Swedish population. Eur Heart J Open. 2023;3:oead024. DOI: 10.1093/ehjopen/oead024 37006408 PMC10063371

[28] Grillo A, Salvi L, Coruzzi P, Salvi P, Parati G. Sodium intake and hypertension. Nutrients. 2019;11:1970. DOI: 10.3390/nu11091970 31438636 PMC6770596

[29] Whelton SP, McEvoy JW, Shaw L, Psaty BM, Lima JAC, Budoff M, et al. Association of normal systolic blood pressure level with cardiovascular disease in the absence of risk factors. JAMA Cardiol. 2020;5:1011–1018. DOI: 10.1001/jamacardio.2020.1731 32936272 PMC7287937

[30] Bild DE, Folsom AR, Lowe LP, Sidney S, Kiefe C, Westfall AO, et al. Prevalence and correlates of coronary calcification in black and white young adults: The coronary artery risk development in young adults (CARDIA) study. Arterioscler Thromb Vasc Biol. 2001;21:852–857. DOI: 10.1161/01.atv.21.5.852 11348886

[31] Taylor AJ, Feuerstein I, Wong H, Barko W, Brazaitis M, O’Malley PG. Do conventional risk factors predict subclinical coronary artery disease? Results from the Prospective Army Coronary Calcium Project. Am Heart J. 2001;141:463–468. DOI: 10.1067/mhj.2001.113069 11231446

[32] Patik JC, Lennon SL, Farquhar WB, Edwards DG. Mechanisms of dietary sodium-induced impairments in endothelial function and potential countermeasures. Nutrients. 2021;13:270. DOI: 10.3390/nu13010270 33477837 PMC7832854

[33] Farquhar WB, Edwards DG, Jurkovitz CT, Weintraub WS. Dietary sodium and health: more than just blood pressure. J Am Coll Cardiol. 2015;65:1042–1050. DOI: 10.1016/j.jacc.2014.12.039 25766952 PMC5098396

[34] Sanders PW. Vascular consequences of dietary salt intake. Am J Physiol Renal Physiol. 2009;297:F237–F243. DOI: 10.1152/ajprenal.00027.2009 19339634 PMC2724242

[35] Joosten MM, Gansevoort RT, Mukamal KJ, Lambers Heerspink HJ, Geleijnse JM, Feskens EJM, et al. Sodium excretion and risk of developing coronary heart disease. Circulation. 2014;129:1121–1128. DOI: 10.1161/CIRCULATIONAHA.113.004290 24425751

[36] Iorga A, Cunningham CM, Moazeni S, Ruffenach G, Umar S, Eghbali M. The protective role of estrogen and estrogen receptors in cardiovascular disease and the controversial use of estrogen therapy. Biol Sex Differ. 2017;8:33. DOI: 10.1186/s13293-017-0152-8 29065927 PMC5655818

