# Supplementary material for: Sodium Intake and Its Relation to Coronary Calcium Score in Relatively Young Korean Adults
Source: Glob Heart. 2026 Feb 16;21(1):10. doi: 10.5334/gh.1528 (PMC12922677; doi:10.5334/gh.1528)
Supplement: Supplementary File. — Supplementary Tables 1 and 2. [file gh-21-1-1528-s1.pdf]

Supplementary Table 1. Baseline clinical characteristics of study participants according to the dietary sodium intake in men.

Characteristics	Quartile 1	Quartile 2	Quartile 3	Quartile 4	P value
number	17,277	17,269	17,269	17,271	
Age (year)	40.3 ± 7.8	40.0 ± 7.6	40.0 ± 7.6	40.3 ± 7.6	< 0.001
BMI (kg/m^2)	24.8 ± 3.0	24.9 ± 3.1	25.1 ± 3.1	25.4 ± 3.2	<0.001
LDL-cholesterol (mg/dl)	132.6 ± 32.0	132.5 ± 31.7	132.1 ± 31.9	131.8 ± 32.0	0.067
Average alcohol use (g/day)	15.3 ± 20.2	17.4 ± 21.9	19.3 ± 24.0	23.1 ± 29.3	<0.001
Current smoker (%)	23.4%	26.7%	29.2%	32.8%	< 0.001
High physical activity (%)	15.6%	16.4%	17.6%	19.1%	<0.001
High education (%)	74.9%	76.6%	76.9%	74.5%	<0.001
Hypertension (%)	17.3%	16.9%	18.2%	18.6%	<0.001
Systolic blood pressure (mmHg)	114.5 ± 11.5	114.6 ± 11.4	115.0 ± 11.4	115.5 ± 11.6	<0.001
Diastolic blood pressure (mmHg)	74.4 ± 9.3	74.4 ± 9.3	74.6 ± 9.3	75.0 ± 9.4	<0.001

Anti-hypertensive medication (%)	7.2%	7.0%	7.7%	7.7%	<0.001
DM (%)	5.6%	5.8%	5.8%	6.3%	0.014
Total calorie intake (kcal/day)	1126.5 ± 361.1	1393.5 ± 406.3	1638.7 ± 467.2	2009.8 ± 627.9	< 0.001
Dietary sodium intake (mg/day)	660.3 ± 236.4	1254.4 ± 157.4	1897.8 ± 225.8	3323.3 ± 962.2	<0.001
Lipid lowering medication use (%)	4.1%	4.2%	4.1%	4.5%	0.157
CAC score	10.9 ± 83.5	11.2 ± 84.5	10.4 ± 71.6	10.1 ± 68.2	0.579
CAC score >0 (n, [%])	2,409 (13.9%)	2,431 (14.1%)	2,463 (14.3%)	2,542 (14.7%)	0.183

Continuous variables are expressed as mean (±SD), and categorical variables are expressed as number (percentage (%)).

BMI: body mass index, DM: diabetes mellitus, LDL: low-density lipoprotein, CAC: Coronary Calcium Score

Supplementary Table 2. Baseline clinical characteristics of study participants according to the dietary sodium intake in women.

Characteristics	Quartile 1	Quartile 2	Quartile 3	Quartile 4	P value
number	5,063	5,063	5,064	5,061	
Age (year)	40.9 ± 9.2	40.9 ± 8.8	41.4 ± 9.2	42.2 ± 9.4	< 0.001
BMI (kg/m^2)	22.2 ± 3.4	22.5 ± 3.4	22.6 ± 3.5	22.9 ± 3.6	<0.001
LDL-cholesterol (mg/dl)	117.9 ± 31.8	118.8 ± 31.9	119.2 ± 32.8	119.2 ± 31.8	0.134
Average alcohol use (g/day)	4.6 ± 9.4	5.3 ± 10.5	5.1 ± 9.6	5.6 ± 11.6	<0.001
Current smoker (%)	1.7%	1.5%	2.0%	1.8%	<0.001
High physical activity (%)	13.5%	15.8%	15.4%	19.2%	<0.001
High education (%)	56.5%	56.2%	54.5%	51.4%	<0.001
Hypertension (%)	7.2%	7.7%	8.5%	9.9%	<0.001
Systolic blood pressure (mmHg)	104.2 ± 11.9	104.6 ± 12.2	104.6 ± 12.1	105.5 ± 12.6	<0.001
Diastolic blood pressure (mmHg)	66.5 ± 8.7	66.8 ± 8.8	66.7 ± 8.9	67.2 ± 9.1	0.003

Anti-hypertensive medication (%)	4.0%	4.1%	5.0%	6.1%	<0.001
DM (%)	3.6%	3.7%	3.6%	4.4%	0.081
Total calorie intake (kcal/day)	936.0 ± 312.7	1159.0 ± 377.4	1371.4 ± 441.7	1732.0 ± 616.9	< 0.001
Dietary sodium intake (mg/day)	586.9 ± 199.9	1094.3 ± 135.1	1658.8 ± 208.0	3072.1 ± 1015.9	<0.001
Lipid lowering medication use (%)	3.2%	3.7%	4.4%	4.4%	0.004
CAC score	4.5 ± 51.2	3.0 ± 30.8	2.8 ± 29.9	5.0 ± 51.7	0.019
CAC score >0 (n, [%])	228 (4.5%)	226 (4.5%)	269 (5.3%)	316 (6.2%)	<0.001

Continuous variables are expressed as mean (±SD), and categorical variables are expressed as number (percentage (%)).

BMI: body mass index, DM: diabetes mellitus, LDL: low-density lipoprotein, CAC: Coronary Calcium Score
